# Characterization of Shiga toxin – producing *Escherichia coli* infections in beef feeder calves and the effectiveness of a prebiotic in alleviating Shiga toxin - producing *Escherichia coli* infections

**DOI:** 10.1186/2046-0481-66-17

**Published:** 2013-10-03

**Authors:** Danica Baines, Stephanie Erb

**Affiliations:** 1 Agriculture and Agri-Food Canada, Lethbridge Research Centre, 5403 1 Avenue South, P.O. Box 3000, T1J 4B1 Lethbridge, AB, Canada

**Keywords:** O157 and non-O157 Shiga toxin- producing *Escherichia coli*, Prebiotic

## Abstract

**Background:**

In the less-sensitive mouse model, Shiga toxin-producing *Escherichia coli* (STEC) challenges result in shedding that reflect the amount of infection and the expression of virulence factors such as Shiga toxins (Stx). The purpose of this study was to characterize the contribution of STEC diversity and Stx expression to shedding in beef feeder calves and to evaluate the effectiveness of a prebiotic, Celmanax®, to alleviate STEC shedding. Fecal samples were collected from calves at entry and after 35 days in the feedlot in spring and summer. STECs were evaluated using selective media, biochemical profile, serotyping and Stx detection. Statistical analysis was performed using repeated measures ANOVA and logistic regression.

**Results:**

At entry, non-O157 STEC were dominant in shedding calves. In spring, 21%, 14% and 14% of calves acquired O157, non-O157 and mixed STEC infections, respectively. In contrast, 45%, 48% and 46% of calves in summer acquired O157, non-O157 and mixed STEC infections, respectively. Treatment with a prebiotic, Celmanax®, in spring significantly reduced 50% of the O157 STEC infections, 50% of the non-O157 STEC infections and 36% of the STEC co-infections (*P =* 0.037). In summer, there was no significant effect of the prebiotic on STEC infections. The amount of shedding at entry was significantly related to the number and type of STECs present and Stx expression (r^2^ = 0.82). The same relationship was found for shedding at day 35 (r^2^ = 0.85), but it was also related to the number and type of STECs present at entry. Stx - producing STEC infections resulted in 100 to 1000 × higher shedding in calves compared with Stx-negative STECs.

**Conclusions:**

STEC infections in beef feeder calves reflect the number and type of STECs involved in the infection and STEC expression of Stx. Application of Celmanax® reduced O157 and non-O157 STEC shedding by calves but further research is required to determine appropriate dosages to manage STEC infections.

## Background

Both O157 and non-O157 STECs are bacteria that cause serious human disease outbreaks through the consumption of contaminated food products [1]. Historically, STEC infections were not linked with the development of disease in older calves or mature cattle [2,3], leading to the conclusion that these bacteria are part of the normal gut flora. Traditional mitigation strategies for preventing STEC transmission to foods were developed based upon the premise that STECs were not pathogens including altering the normal flora using direct-fed microbial feed additives [4] and procedural changes at slaughter [5]. Recent studies indicate that STEC co-infections and mycotoxins are part of the disease complex for Jejunal Hemorrhage Syndrome (JHS) in beef feeder calves and mature dairy cattle [6,7] supporting an interaction between the host and pathogen that leads to infection. Several other studies provide indirect evidence that supports the hypothesis that STECs interact with the intestinal tract of older age classes of cattle [8-15]. The clinical symptoms described for STEC infections in beef feeder calves and mature cattle resemble those described in another less sensitive STEC disease model, the mouse, and include discolored feces, hind-limb paralysis, tremors, ataxia and death [8-10]. Persistent shedding in beef cattle is associated with repeated isolations of challenge STEC from experimental cattle [11] and repeated isolations of STEC clones from natural infections in the feedlot [12]. Stx is associated with STEC infection in challenge studies with immature calves [13-15] and Stx2 increases STEC colonization of enterocytes isolated from mature cattle *in vitro*[16] while, Stx1 is toxic to particular classes of lymphocytes in immature calves [15]. If we are to improve on current methods to reduce food safety concerns associated with STEC transmission to foods, it is essential that we examine STEC pathogenesis in older calves and mature cattle. For example, what role if any do Stxs play in promoting infections and shedding [17], are other pathogens present [18] and what is the nature of the infections in different shedding categories [19]. Using the mouse model as a guide, shedding patterns and susceptibility to disease are related to STEC virulence traits with the majority of avirulent strains producing transient infection and shedding with no symptoms [8-10]. As antibiotics are contraindicated for STEC infections in humans [20] and prebiotic/probiotic applications alleviate the clinical symptoms and the development of acute STEC-associated disease in cattle [6,7], a similar approach could address food safety concerns. The first objective of the current study was to characterize STEC pathogenesis in beef feeder calves with respect to the source of the infections, the type of STECs involved and the role of Stxs. The second objective was to determine the impact of a prebiotic, Celmanax®, application on STEC infections and shedding in beef feeder calves*.*

## Methods

The protocols were reviewed by the Agriculture and Agri-Food Canada Animal Care Committee and approved under ACC protocol 1131. This study had two main objectives, first to characterize STEC infections in beef feeder calves and second, to determine the impact of a prebiotic on STEC shedding.

### Experimental animals

Two groups of heifers were used in the study: LOAD 1 with 56 animals and an average body weight of 193 kg; and LOAD 2 with 63 animals and an average body weight of 189 kg. The animals arrived in Texas on May 5, 2011 and June 16, 2011 respectively and the heifers were randomly assigned to treatments. The receiving diet consisted of 0.9 kg/heifer of ryegrass hay and water with or without the treatment, Celmanax® (14 g/[heifer•d]. The heifers were processed and assigned to one of 12 pens. The heifers were provided either a control diet consisting of 65% concentrate with the major ingredients steam-flaked corn, alfalfa hay, cottonseed hulls, cottonseed meal, molasses, and animal-vegetable fat or the same diet supplemented with Celmanax® (14 g/[heifer•d]. The Celmanax® consists of a non-living formulation of yeast cell walls or mannan oligosaccharide (MOS) and yeast metabolites. This product has been shown to antagonize O157 and non-O157 STEC colonization and eliminate feed-associated mycotoxin cytotoxicity *in vitro*[6,7]. To evaluate STEC infections in the calves, fecal ESWAB samples (Alere™, Ottawa, Ontario Canada) were collected during the weighing process on day 0 and day 35.

### STEC characterization

Fecal samples from the control and treatment heifers were evaluated for O157 and non-O157 STECs using a method that was developed previously [6,7]. Fecal samples were weighed (0.01 g per swab) and all samples were glycerol stocked (20%) and stored at –20°C or serial dilutions were applied to CHROMagar™ O157 plates (Dalynn Biologicals, Calgary, Alberta). Using this approach, STECs are isolated from CHROMagar™ O157 with O157 STEC appearing as mauve colonies and non-O157 STEC appearing as mauve colonies or blue colonies with a mauve halo. To confirm identity, the presumptive isolates were subjected to a GN-ID A + B biochemical test (Alere™, Ottawa, Ontario, Canada). To distinguish potential O157 STECs from non-O157 STECs, bacteria were plated onto Cefixime-Tellurite Sorbitol MacConkey agar (CT-SMAC; Dalynn Biologicals, Calgary, Alberta, Canada) to identify non-sorbitol and sorbitol fermenting bacterial colonies. All suspect colonies were tested as O157 and H7 using the RIM™ E. coli O157 latex test (Fisher Scientific, Ottawa, Ontario, Canada). To further characterize the composition of STEC infections, presumptive STECs were also tested using the Remel Polyvalent 2 (O26, O55, O111, O119, O126) sera, Remel Polyvalent 3 (O83, O114, O125, O127, O128) sera and Remel Polyvalent 4 (O44, O112, O124, O142) sera (Oxoid, Nepean, Ontario, Canada). Because O26 and O111 STEC infections are on the rise in human disease outbreaks, all STECs positive for polyvalent 2 were subsequently tested using Denka-Seiken O26 and O111 monovalent sera (Oxoid, Nepean, Ontario, Canada). All bacterial colonies that were identified as *E. coli* in the GN-ID A + B test were characterized for Stx1 and Stx2 expression using an ImmunoCard STAT!® EHEC test (Somagen, Edmonton, Alberta, Canada) [6,7].

The statistical analysis was performed using isolates that were biochemically defined as *E. coli* and identified to serotype.

### Statistical analysis

Statistical analyses were conducted utilizing Repeated Measures ANOVA for evaluating the impact of the prebiotic on STEC shedding (SYSTAT 10.2.01). The statistical model used for the analysis was yijt = yo + μ + di + γj(i) + τt + (dτ)it + eijt: yijt is the measurement taken at time t on the jth calf assigned to the ith diet treatment, yo is a covariate (pre-treatment measurement), μ is the overall mean effect, di is the ith fixed diet effect, γj(i) is the random effect of the jth calf within ith diet treatment, τt is the fixed time effect when the measurement was taken, (dτ) it is the fixed interaction effect between diet and time, eijt is the random error associated with the jth calf assigned to the ith diet treatment at time t.

Analysis was also performed using multinomial logistic regression to evaluate the contribution of STEC identity, the number of STECs present and Stx expression on infection as evidenced by shedding at entry and after 35 days in the feedlot.

Results were considered significant if *P* < 0.05 and non-significant if *P* > 0.05.

## Results

### Randomizing calves to treatments

Randomizing the calves to pens did not result in an even distribution of STEC-shedding calves to treatments. The control treatments for spring had 76% non-shedding calves and 24% shedding calves. In contrast, the prebiotic treatments for spring had 61% non-shedding calves and 39% shedding calves. Similarly, the control treatments for summer had 64% non-shedding calves and 36% shedding calves. In contrast, the prebiotic treatments for summer had 46% non-shedding calves and 54% shedding calves. More critically, there was a higher percentage of moderate to high shedding calves at entry in spring and summer in the Celmanax® treatment (spring, 32%; summer 19%) compared with the control treatment (spring, 11%; summer, 10%). The results from the current study suggest that these imbalances could influence the evaluation of the effectiveness of the treatment and thus, more effort must be taken at entry to balance the shedding calves across treatments.

### STEC identification

The method used in the current study is a novel approach for detecting STECs in calves thereby, an explanation of the STEC morphologies and other false positive pathogenic Enterobacteriaceae are provided. Non-STEC pathogens appeared as large mauve colonies with a white halo and an irregular edge or yellow regular smooth colonies with a regular edge. These isolates were confirmed as pathogenic bacteria in the GN-ID A + B test. Less than 5% of the animals in the study were co-infected with *Citrobacter* species and *E. fergusonii*, but these animals were associated with low STEC shedding rates (<10^2^ CFU/0.01 g feces) and were not detected in the 35 day sample. This suggests that co-infection with other Enterobacteriaceae does not contribute to STEC infections in beef feeder calves.

Presumptive STECs had two phenotypes on CHROMagar™ O157 plates: mauve colonies with a white halo that had a regular or irregular edge (O157 STEC and non-O157 STEC); and navy blue colonies with a mauve halo (non-O157 STEC) that had a regular or irregular edge. There was a high degree of variation in the blue colonies with mauve halos that distinguished one non-O157 STEC from another. Presumptive STECs were confirmed as pathogenic *E. coli* in the GN-ID A + B test, serotyped and the Stx expression profile determined. At entry to the feedlot in spring, 70 potential STECs were isolated and 24 STECs were confirmed (Table 1). The false positive isolates were collected at 48 hr suggesting that evaluations performed after 24 hr are invalid. After 35 days in the feedlot, 93 potential STECs were isolated and 58 STECs were confirmed (Table 1). At entry to the feedlot in summer, 70 potential STECs were isolated and 32 STECs were confirmed. After 35 days in the feedlot, 120 potential STECs were isolated and 100 STECs were confirmed (Table 1). The prevalence for O157 STEC shedding by beef feedlot calves using this CHROMagar™ O157 detection method is within the range reported for beef feeder calves in feedlot environments in the USA using PCR and IMS – based methods [21,22]. In September 2011, the Food Safety and Inspection Service in the USA declared 6 non-O157 STECs adulterants including serotypes O26, O111, O103, O121, O145 and O45 [23]. The prevalence for common non-O157 STEC serotypes in beef feeder calves were determined in this study and are provided in Table 2. These prevalence rates reflect those reported for beef feedlots in the USA using PCR and IMS – based detection systems [21,22]. There were 2 calves that had single isolate infections with non-O157 STEC displaying the auto-agglutinating adhesin described for LEE-negative STECs [24]. One strain produced Stx1 and the other strain produced Stx2. After 35 days in the feedlot in spring, there was an increase in the prevalence of O111 STEC, other STEC within the polyvalent 2 sera group (O55, O119, O126) and polyvalent 3 sera group (O83, O114, O125, O127, O128). There were no increases in the O26 or polyvalent 4 sera group (O44, O112, O124 and O142). After 35 days in the feedlot for summer, the same increase in O111 STEC, polyvalent 2 sera group and polyvalent 3 sera group was detected. However, there was a decline in 026 STEC shedding.

**Table 1:** Prevalence of O157 and non-O157 STECs in beef feeder calves in spring and summer

	**Percent O157 STEC**		**Percent Non-O157 STEC**	
**Season**	**Day 0**	**Day 35**	**Day 0**	**Day 35**
**Spring (n = 56)**	7	27	36	77
**Summer(n = 61)**	11	87	40	72

**Table 2:** Prevalence of non-O157 STEC serotypes shed by beef feeder calves in spring and summer [polyvalent 2 sera (O26, O55, O111, O119, O126), polyvalent 3 sera (O83, O114, O125, O127, O128), polyvalent 4 sera (O44, O112, O124 and O142), monovalent O26 sera and monovalent O111 sera]

	**Percent non-O157 STEC**				
**Season**	**P2**	**O26**	**O111**	**P3**	**P4**
**Spring day 0**	3	17	17	3	7
**Spring day 35**	14	17	39	39	7
**Summer day 0**	6	19	12	0	6
**Summer day 35**	15	3	46	25	3

The current study together with previous studies [6,7] suggest that the CHROMagar™ O157 method in combination with biochemical and serotype testing is effective in isolating O157 and non-O157 STECs shed in the feces of calves in feedlots.

### Seasonal STEC shedding

**Figure 1:**
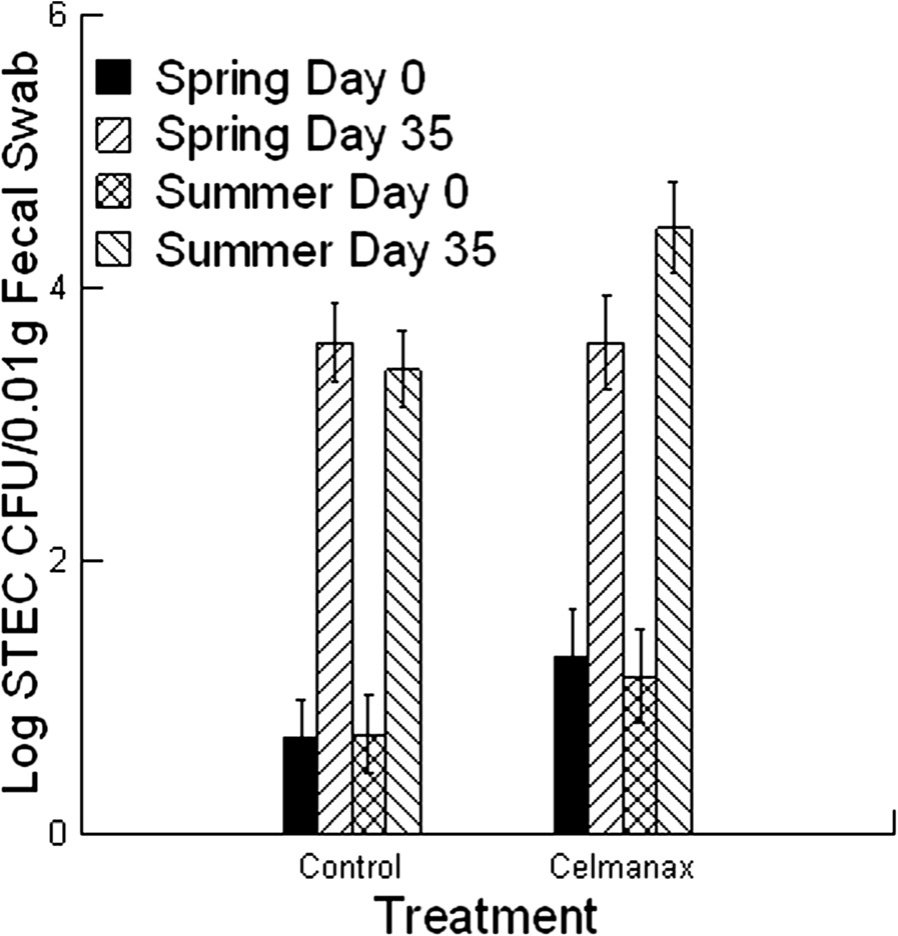
**The average number of Shiga toxin – producing *Escherichia coli *(STEC) shed by beef feeder calves fed diets containing 0 (control) or 14 g/[heifer•d] Celmanax**^**®**^**.** The fecal samples were collected on day 0 and day 35. Mean values are shown (n = 28 for both treatments in spring; n = 30 for control treatments in summer; n = 31 for Celmanax^®^ treatments in summer).

The average STEC shedding by calves was significantly greater in summer compared with spring (Figure 1; *P =* 0.001). This change was associated with a higher percentage of calves shedding STEC at entry and day 35. For the control treatment at entry, the percentage of calves shedding STEC was 25% in spring and 36% in summer. For the control treatment at day 35, the percentage of calves shedding STEC was 64% in spring and 82% in summer. For the Celmanax® treatment at entry, the percentage of calves shedding STEC was 39% in spring and 55% in summer. For the Celmanax® treatment at day 35, the percentage of calves shedding STEC was 64% in spring and 93% in summer. The seasonal changes in shedding patterns for STEC in the control calves indicated that most infections were acquired in the feedlot (60-70%) while some infections identified at entry (10%) were maintained over the 35 day period. Interestingly, about 20% of the control calves did not enter the feedlot with STEC infections or acquire infections during the study period.

### Seasonal O157 STEC, non-O157 STEC and mixed STEC shedding

**Figure 2:**
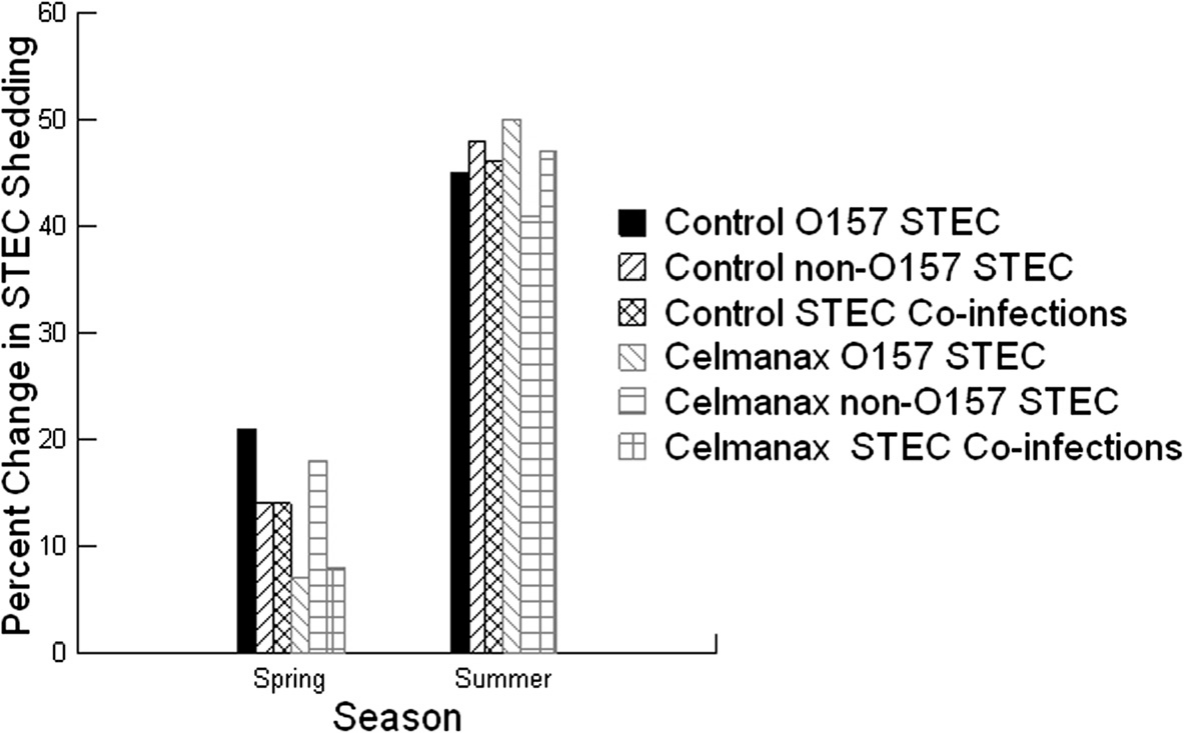
**The percentage change in the amount of Shiga toxin – producing *Escherichia coli *(STEC) shed between day 0 and day 35 by beef feeder calves fed diets containing 0 (control) or 14 g/[heifer•d] Celmanax**^**®**^**.** The fecal samples were collected at day 0 and day 35. Mean values are shown (n = 28 for both treatments in spring; n = 30 for control treatments in summer; n = 31 for Celmanax^®^ treatments in summer).

The composition of STEC infections significantly changed over the 35 day period in the feedlot (*P =* 0.001, Figure 2).The percentage of calves shedding STECs changed over the experimental period and is an expression of maintained, non-maintained or new STEC infections. In the control treatment at entry in spring, O157 STEC infections were all newly acquired and contributed to 21% of calves shedding after 35 days. In contrast, non-O157 STEC infection (21%) in calves at entry decreased to 14% due to non-maintained, maintained or new infections after 35 days. Infections with STEC mixtures consisting of O157 and non-O157 STEC (7%) at entry increased to 14% due to maintained or new infections after 35 days. In the control treatment in summer, O157 STEC infection (10%) in calves at entry increased to 46% due to maintained or new infections after 35 days. Similarly, non-O157 STEC infection (23%) in calves at entry in summer increased to 27% due to maintained or new infections after 35 days. Infections with STEC mixtures (7%) in calves at entry in summer increased to 35% due to maintained or new infections after 35 days. These results indicate that stressor events such as heat that occur in feedlots promote acquisition and growth of STEC infections in calves.

In the Celmanax® treatment in spring, O157 STEC infection (14%) in calves at entry decreased to 7% due to non-maintained or maintained infections after 35 days. In contrast, non-O157 STEC infections (28%) in calves decreased to 14% due to non-maintained or maintained infections after 35 days. Infections with STEC mixtures (11%) in calves at entry in spring decreased to 7% due to non-maintained or maintained infections after 35 days. In the Celmanax® treatment in summer, O157 STEC infection (13%) in calves at entry increased to 52% due to maintained or new infections after 35 days. In contrast, non-O157 STEC infections (35%) in calves increased to 42% due to maintained and new infections after 35 days. Infections with STEC mixtures (6%) in calves in summer increased to 48% due to maintained or new infections after 35 days.

The results suggest that the application of the 14 g/[heifer•d] of Celmanax® reduced entry O157 STEC infections and prevented the acquisition of new infections in calves supporting previous in vitro studies which showed anti-O157 STEC colonization properties for the prebiotic [6,7]. Despite having a higher percentage of entry non-O157 STEC infections in calves compared with control treatments, the Celmanax® also reduced entry non-O157 infections and the acquisition of new infections in calves supporting previous in vitro studies which showed anti-non-O157 STEC colonization properties for the prebiotic [6,7]. Similarly, the Celmanax® treatment reduced entry STEC co-infections and the acquisition of new co-infections in calves. This was recorded as a lower conversion of either non-shedding calves to shedding calves or low and moderate shedding calves to high shedding calves. The results for summer were not significant, but there was a similar trend as spring where the application of the 14 g/[heifer•d] of Celmanax® reduced O157 and non-O157 STEC infections and the conversion of low and moderate shedding calves to high shedding calves.

### STEC diversity and shedding by calves

**Figure 3:**
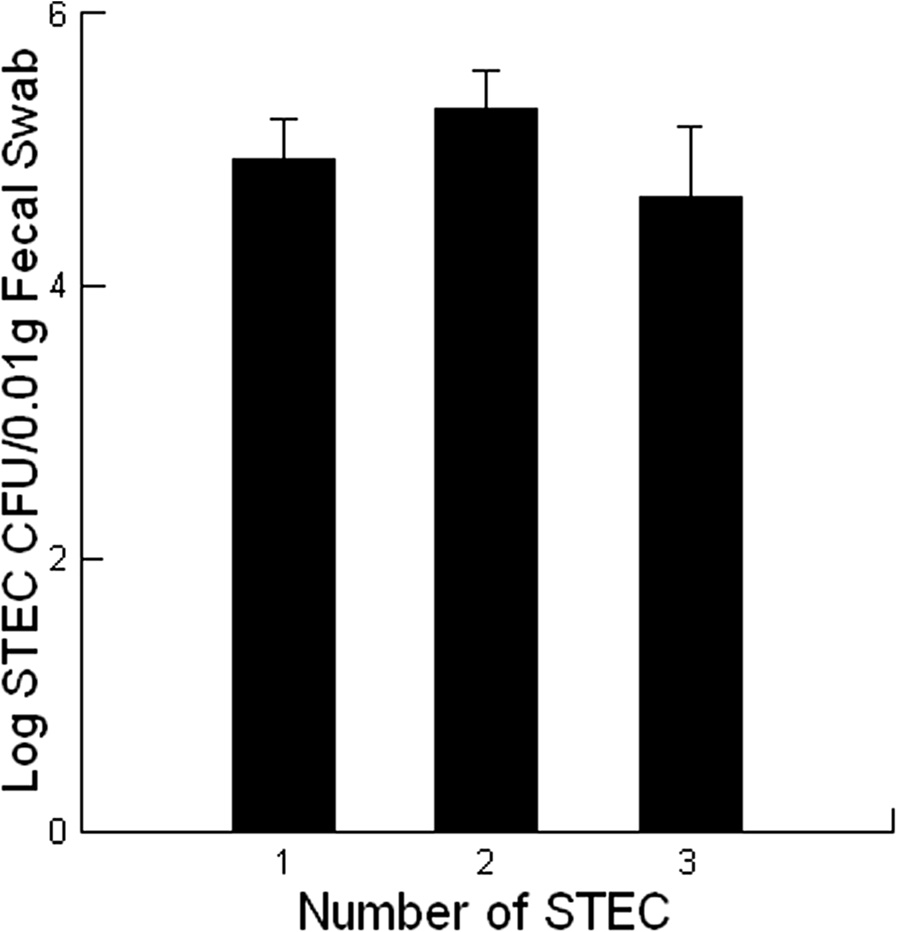
**The effect of the number of Shiga toxin – producing *Escherichia coli *(STEC) involved in an infection on the amount of STEC shed by beef feeder calves fed diets containing 0 (control) or 14 g/[heifer•d] Celmanax**^**®**^**.** The fecal samples were collected at day 0 and again at day 35. Mean values are shown (n = 28 for both treatments in spring; n = 30 for control treatments in summer; n = 31 for Celmanax^®^ treatments in summer). The diversity of STECs was scored as follows: 1 = one STEC in the fecal sample; 2 = two STEC in the fecal sample, 3 = more than 2 STEC in the fecal sample).

Many experimental challenge studies have been performed with beef feeder calves using inoculants containing one or more O157 STEC [2,3,13,14]. However, these studies have not examined whether other STEC infections already present in the calves are contributing to the successful infection with the challenge STEC. In the current study, shedding at day 35 was predictable based on the number of STECs involved in the infection (*P =* 0.001, Figure 3) which indicated that co-infection increased the likelihood of shedding by calves. The greater variability in the shedding at day 35 for calves with two or more STECs involved in the infection suggest that there may be an interaction between these pathogens that dynamically changes as virulence factors are expressed.

### O157 STEC, Stx expression and shedding by calves

**Figure 4:**
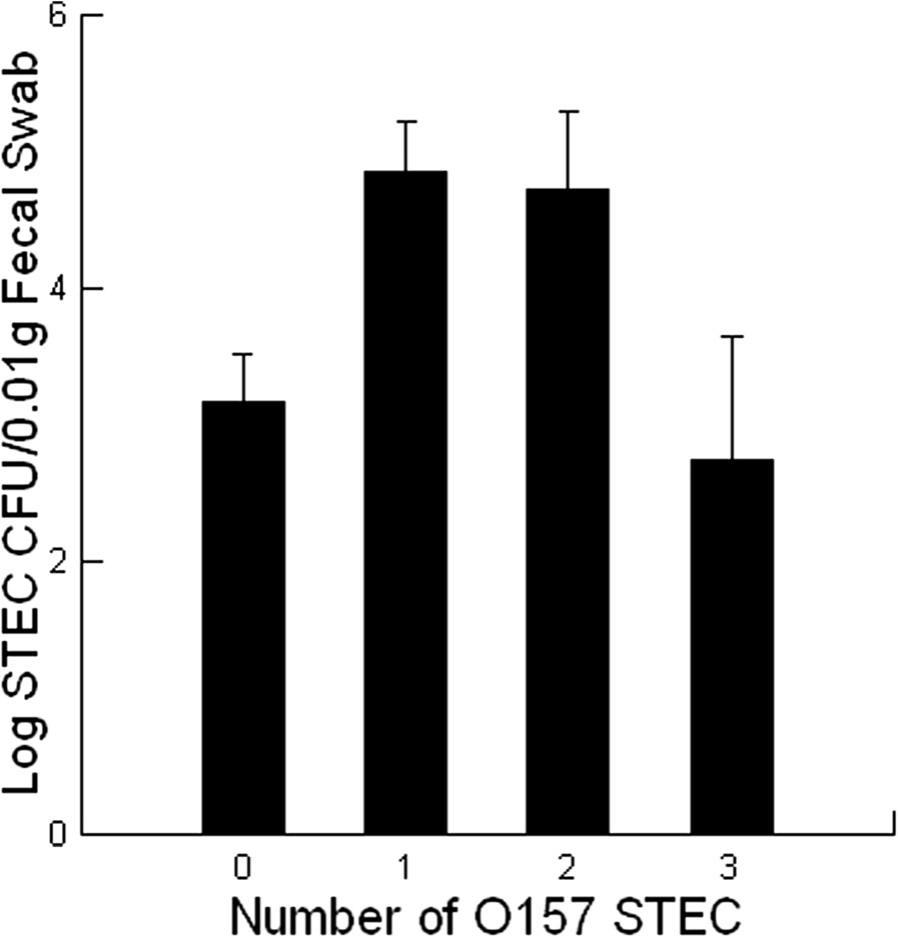
**The effect of the number of O157 Shiga toxin – producing *Escherichia coli *(STEC) involved in an infection on the amount of STEC shed by beef feeder calves fed diets containing 0 (control) or 14 g/[heifer•d] Celmanax**^**®**^**.** The fecal samples were collected at day 0 and day 35. Mean values are shown (n = 28 for both treatments in spring; n = 30 for control treatments in summer; n = 31 for Celmanax^®^ treatments in summer). The contribution of O157 STEC infections was scored as follows: 1 = one O157 STEC in the fecal sample; 2 = two O157 STECs in the fecal sample, 3 = greater than two O157 STECs in the fecal sample.

**Figure 5:**
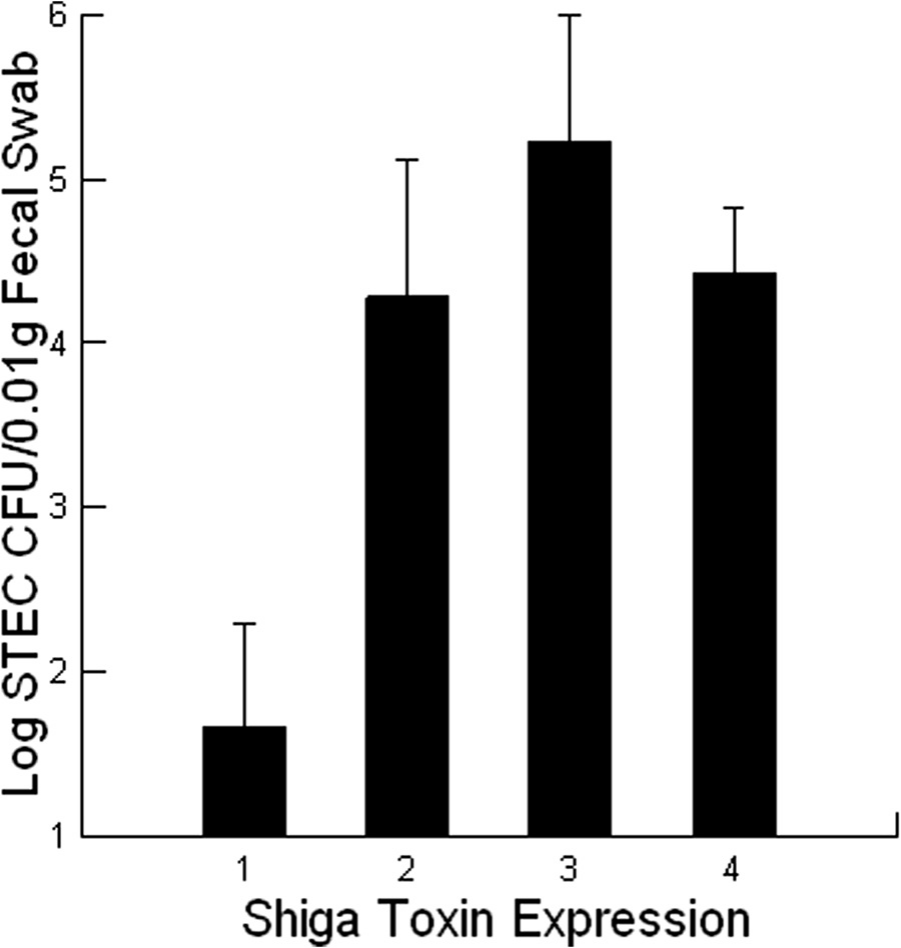
**The effect of Shiga toxin (Stx) expression on the amount of Shiga toxin – producing *Escherichia coli *(STEC) shed by beef feeder calves fed diets containing 0 (control) or 14 g/[heifer•d] Celmanax**^**®**^**.** The fecal samples were collected at day 0 and again at day 35. Mean values are shown (n = 28 for both treatments in spring; n = 30 for control treatments in summer and n = 31 for CelmanaxTM treatments in summer). The contribution of Stx expression was scored as follows: 1 = no Stx expression; 2 = Stx1 expression; 3 = Stx2 expression; 4 = Stx1 and Stx2 expression.

Shedding rates after 35 days in the feedlot occurred when at least one O157 STEC was part of the infection (*P =* 0.011, Figure 4). Interestingly, when there were three or more O157 STECs present the shedding patterns were lower and resembled non-O157 STEC infections. This suggested that the virulence traits for STECs may potentially be driving successful infection and shedding in calves rather than serotype. One virulence trait that is critical for establishing STEC infections in humans and mice is Stx production [1]. For example, a challenge with Stx2 – producing strains is associated with establishing high infections that result in the development of clinical symptoms and disease in neonatal calves [13,14] and mice [8-10]. Herein we describe a similar feature where the expression of Stxs causes a significant 100 to 1000 times greater infection as evidenced by shedding when compared with STECs not expressing Stxs (*P =* 0.001, Figure 5). Significantly, Stx2 – producing STEC infections consistently provided the highest shedding in the calves and in this way resembles results obtained with the less sensitive mouse model [1].

## Discussion

The current study demonstrates that beef feeder calves show enhanced susceptibility to STEC infection and shedding in response to Stx-producing strains as assessed by fecal shedding. In addition, STEC mixtures and more specifically, those infections associated with O157 STECs were required to elicit high shedding.

Experimental challenge studies in mature cattle are performed using from one to four STECs with each producing Stx1 and Stx2 [2,3,11,13,14]. The STECs used in challenge studies for the most part originate from human disease outbreaks rather than calf disease outbreaks. Overall, this approach may have contributed to our lack of understanding of how STEC virulence traits affect different age classes of cattle. Mature cattle respond to STEC challenges with low, moderate and high shedding rates with the magnitude and duration affected by age [2,3], exposure dose [2] and a mucosal factor [25]. Typically, the results of these challenge studies are presented as a total count per gram feces which represent the challenge STEC(s). Therefore, it is impossible to determine if there was one or more challenge or non-challenge STECs contributing to the shedding rates. However, these studies do suggest that persistent shedding or high infection can be achieved using multiple O157 STECs [11,25]. There is also some evidence to support strain origin or virulence traits as contributing to infection and severity of disease in neonatal calves [13]. For example, challenging neonatal calves with an O157 STEC (3081, pig origin) caused greater clinical symptoms and pathology than O157 STEC (EDL-933, human origin) including diarrhea, colonic edema, A/E lesions, diffuse neutrophil infiltration, diffuse atrophy of ileal villi and fibrinohemorrhagic pseudomembranes. The current study extends this information and indicates that STEC diversity and at least one virulence trait, Stx expression, contribute to the severity of infection and subsequent shedding by beef feeder calves.

There have been many studies examining the prevalence and persistence of O157 or non-O157 STEC in feces of beef feeder calves in feedlots [21,22]. Repeated isolations of genetically distinct STECs from feedlots has suggested that the main source for STEC transmission was the feedlot environment and not the incoming calves [12]. The current study confirmed that about 60% of STEC infections that result in shedding occur as a result of newly acquired STEC infections in the feedlot. There was, however, a noticeable 10% increase in the proportion of control calves shedding O157 STEC at entry in summer compared with spring. Unlike the non-O157 infections, the O157 STEC infections grew and developed into co-infections with O157 and non-O157 STECs resulting in higher shedding. This suggests that the origin of the O157 STEC challenge within the feedlot is critical for understanding how to interfere with the infection process. A clue to the higher O157 STEC shedding at entry in summer may derive from the calves experience in the calf rearing facility. If we look back in time, the calves in the current study would have been less than a month old in December or January and been transported to the current feedlot in May and June respectively. In Alberta, this time period coincides with higher incidences of *E. coli* scours in calves and seasonal increases in moldy feeds [6,7]. These factors contribute to the development of STEC – associated JHS cases in beef feeder calves [7] and coincide with higher prevalence of O157 STEC in feedlot environments [26]. Therefore, early calf exposure to STEC infections coupled with moldy feeds or stressor events such as transport [27] could result in the suppression of the cellular and humoral immune response in calves [28,29] leading to chronic STEC infections which then, could carry over to the next feedlot and account for seasonal variation in shedding patterns by calves.

Most STECs that are associated with the development of HUS in humans do not cause clinical symptoms or disease in mice, but there are a few isolates with unique virulence factors that enable the STEC to colonize the intestine and produce mucosal damage that facilitates the development of systemic disease [8-10]. The primary virulence factor associated with high colonization is Stx production in humans and mice with Stx2 production associated with more severe clinical symptoms [1,8]. The current study suggests that Stx promotes STEC infection in beef feeder calves. For example, 10 times greater shedding was achieved for STEC expressing Stx2 alone compared with STEC expressing Stx1 or both Stx1 and Stx2. The role of Stx1 in the STEC infection process in calves is unclear, but it may increase infection by preventing cellular immune responses [15]. The role of Stx2 in the STEC infection process has been examined in STEC challenge studies with neonatal calves. STEC containing the *eae* and *stx2* genes achieved greater clinical symptoms and development of disease compared with *eae*- or *stx2-* negative strains [13]. This agrees with *in vitro* studies which suggested that Stx2 promoted higher STEC (bovine or human origin) colonization of the mucosa and a bovine colonic cell line [16]. More significantly, STEC mixtures expressing one or more Stxs caused higher infections that led to greater fecal shedding. The similarity between beef feeder calf and neonatal calf responses to natural STEC challenges support the hypothesis that older calves are developing STEC infections and are not simply reservoirs.

Beef feeder calves encounter a variety of stressor events such as extreme weather conditions during transport to and after entry into the feedlot environment [27,29]. Such stressor events are known to reduce the ability of calves to fight pathogen infections which can lead to the development of chronic scours [30,31]. The Celmanax® application did prevent: 1) the maintenance and acquisition of new O157 and non-O157 STEC infections in calves; and 2) the maintenance and acquisition of new STEC co-infections in spring, but was unable to do so effectively in summer. This suggests that the dosage was insufficient under summer feedlot conditions possibly due to a greater number of stressor events present in the feedlot environment.

## Conclusions

This study conducted on beef feeder calves supports a role for STEC diversity and Stx expression in influencing severity of STEC infection and shedding by calves. In spring, application of 14 g/[heifer•d] Celmanax® was effective at reducing the maintenance and acquisition of single and mixed STEC infections in calves, but was ineffective in summer. Further studies are required to determine the effective dosage of Celmanax® to manage STEC shedding in calves throughout the year.

## Competing interests

Both authors declare that they have no competing interests.

## Authors’ contributions

DB designed the study, analyzed the fecal samples, performed the statistical analysis and drafted the manuscript; SE analyzed the fecal samples. Both authors read and approved the final manuscript.
